# Modest Rise in Caesarean Section from 2000-2010: The Dutch Experience

**DOI:** 10.1371/journal.pone.0155565

**Published:** 2016-05-18

**Authors:** Yanjun Zhao, Jun Zhang, Chantal Hukkelhoven, Pien Offerhaus, Joost Zwart, Ank de Jonge, Caroline Geerts

**Affiliations:** 1 MOE-Shanghai Key Laboratory of Children’s Environmental Health, Xinhua Hospital,Shanghai Jiao Tong University School of Medicine, Shanghai, China; 2 The Netherlands Perinatal Registry, Utrecht, the Netherlands; 3 AVM (Academy for Midwifery Maastricht), Maastricht, the Netherlands; 4 Department of Obstetrics and Gynaecology, Deventer Ziekenhuis, Deventer, the Netherlands; 5 Midwifery Science, AVAG/EMGO, VU University Medical Centre, Amsterdam, the Netherlands; University of Barcelona, SPAIN

## Abstract

**Background:**

The caesarean delivery (CD) rate has risen in most countries over the last decades, but it remains relatively low in the Netherlands. Our objective was to analyse the trends of CD rates in various subgroups of women between 2000 and 2010, and identify the practice pattern that is attributable to the relative stability of the Dutch CD rate.

**Methods:**

A total of 1,935,959 women from the nationwide Perinatal Registry of the Netherlands were included. Women were categorized into ten groups based on the modified CD classification scheme. Trends of CD rates in each group were described.

**Results:**

The overall CD rate increased slightly from 14.0% in 2000–2001 to 16.7% in 2010. Fetal, early and late neonatal mortality rates decreased by 40–50% from 0.53%, 0.21%, 0.04% in 2000–2001 to 0.29%, 0.12%, 0.02% in 2010, respectively. During this period, the prevalence of non-vertex presentation decreased from 6.7% to 5.3%, even though the CD rate in this group was high. The nulliparous women with spontaneous onset of labor at term and a singleton child in vertex presentation had a CD rate of 9.9%, and 64.7% of multiparous women with at least one previous uterine scar and a singleton child in vertex presentation had a trial of labor and the success rate of vaginal delivery was 45.9%.

**Conclusions:**

The Dutch experience indicates that external cephalic version for breech presentation, keeping the CD rate low in nulliparous women and encouraging a trial of labor in multiparous women with a previous scar, could help to keep the overall CD rate steady.

## Introduction

There has been much concern about the increasing rate of caesarean delivery (CD) in most high-resource countries over the past decades. The potential explanation for this progressive trend is multifaceted, including changing characteristics of women and her pregnancy such as increasing maternal body mass, delayed childbearing, more multiple gestations, and a lower rate of vaginal birth after previous cesarean (VBAC)[1,2]. Socio-cultural factors also contribute to the rising CD rate such as maternal request[3], socio-cultural attitudes[4], and the obstetricians’ fear of litigation[5]. However, a few countries, such as the Netherlands, are the exceptions where the CD rate has remained relatively low and almost stable in the last decade [6–9]. Their CD practice may give us a unique opportunity to understand how to maintain a relatively low CD rate while continuing to improve perinatal outcomes. This information may be useful in implementing strategies to optimize CD practices in other countries.

The lack of a reliable and standard classification scheme has hampered attempts to audit and analyze underlying causes of the rising CD trends[10,11]. To this end, the Robson ten group classification scheme[10,11] was developed in 2001. It categorizes CD according to the characteristics of each woman and her pregnancy. It has gained increasing acceptance by the obstetric community. However, after being in use for over a decade, suggestions to improve the classification scheme have been proposed[12,13]. For example, several investigators recommended to separate labor induction from prelabor CD[13]. Recently, Zhang et al. suggested to combine three non-cephalic groups into one to keep the total groups to 10 since breech vaginal birth is no longer promoted in many countries and because of the low numbers for non-cephalic presentations[12]. Furthermore, in the Robson classification scheme subgroups are labeled as one to ten. It is challenging to recognize all these figures for their specific meaning. We, therefore, propose a new labeling scheme using only two letters to make the group label more intuitive (Table 1).

**Table 1:** Comparison between the Robson and the modified classification scheme.

Group label	Robson Classification group No.	Characteristics of the group
NS	1	**Nulliparous** women with a single vertex pregnancy, at ≥37 weeks gestation in **spontaneous** labor
NI	2a	**Nulliparous** women with a single vertex pregnancy, at ≥37 weeks gestation who had labor **induced**
NC	2b	**Nulliparous** women with a single vertex pregnancy, at ≥37 weeks gestation, who had **caesarean** section before labor
MS	3	**Multiparous** women, without a uterine scar, with a single vertex pregnancy at ≥37 weeks gestation in **spontaneous** labor
MI	4a	**Multiparous** women, without a uterine scar, with a single vertex pregnancy at ≥37 weeks gestation, who had labor **induced**
MC	4b	**Multiparous** women, without a uterine scar, with a single vertex pregnancy at ≥37 weeks gestation, who had **caesarean** section before labor
PC	5	Multiparous women, with at least one **previous caesarean** section with a single vertex pregnancy at ≥37 weeks gestation
BR	6+7+9	All women with a single **breech**, transverse or other abnormal fetal presentation, including women with a uterine scar
TW	8	All women with multiple pregnancies (e.g., **twins**), including women with a uterine scar
PT	10	All women with a single vertex pregnancy at ≤36 weeks gestation (i.e., **preterm**), including women with a uterine scar
UK		All women who cannot be classified due to **unknown** characteristics

The aims of this study are to (1) describe the CD rate over a recent decade in the Netherlands based on the modified classification scheme; (2) identify the underlying factors in CD practice associated with the relatively low CD rate by examining the change in CD rate in different maternal age, gestational age and birthweight categories.

## Materials and Methods

### The Dutch data

We used data from the national database, the Perinatal Registry (‘Perinatale Registratie–PR’, previously called PRN) of the Netherlands. Data on antenatal, intrapartum and postnatal care are available in three separate databases: primary midwife-led care (LVR1), obstetrician-led care (LVR2), and neonatal care (LNR). The three databases are combined via a validated linkage method[14]. PR contains approximately 95% of all births in the Netherlands[15].

In this study, we selected records of births at a gestational age from 28 weeks + 0 days to 44 weeks + 6 days for the period 2000 to 2010. Given the importance of mode of birth in our analysis, we excluded the birth records that contained no information on mode of birth. We analysed data for infant characteristics on birth data, and maternal characteristics on pregnancy data. In case of a multiple pregnancy, we selected the record of the second baby of multiplets to identify the maternal characteristics.

### Definition of variables

The recorded characteristics consisted of maternal age, ethnicity, parity, socioeconomic status (SES). Given that there are no linear relationship between the outcome and some continuous variables (such as birthweight, gestational age), we choose certain categories and not describe them as continuous variables. Maternal age was categorized into four groups: <20, 20–29, 30–34,≥35 years. SES was derived from social status scores based on an available four digits postal code, developed by the National Institute for Social Research which takes into account the level of education, employment, and income[16]. SES was classified as low (below 25th percentile), medium (between 25th and 75th percentile), and high (above 75th percentile). Given that the ethnic background recorded in PR was not categorized precisely and may not always be consistent in the various non-Dutch categories, we classified ethnicity as Dutch or non-Dutch[16]. Pregnancy related variables included parity (nulliparous and multiparous women), past CD (none, one or more), gestational age at birth (28–31, 32–36, ≥37 weeks), fetal presentation (vertex, non-vertex), number of fetuses (singleton, multiple) and infant birth weight (<2500, 2500–4000, >4000 g).

### Data analysis

We first described the characteristics and perinatal outcomes of all births/pregnancies from 2000 to 2010. We then calculated the CD rates in the 10 groups of the modified Robson classification system. The main outcomes of this analysis were the CD rate for each group, and its contribution to the overall CD rate. Finally, we examined the change in CD rate over time in different maternal age, birthweight and gestational age. All analyses were performed with the statistical software package SPSS 20.0 (SPSS Inc, Chicago, IL, USA).

## Results

From 2000 to 2010, the PR data contained 1,940,649 pregnancies with 1,977,853 newborns in total with a gestational age from 28 weeks + 0 days to 44 weeks + 6 days. We excluded 4690 (0.03%) pregnancies that contained no information on mode of birth, leaving 1,935,959 pregnancies for analysis.

Table 2 shows the characteristics of all births/pregnancies. The maternal age over 35 years increased from 17.8 percent in 2000–2001 to 21.6 percent in 2010. The proportion of non-Dutch women increased from 18.4 to 22.4 percent. The percentage of multiple gestations decreased from 2.0 to 1.6 percent of all pregnancies over that decade. The prevalence of non-vertex presentation changed from 6.7% to 5.3%. The overall CD rate increased slightly from 14.0% in 2000–2001 to 16.7% in 2010 with the primary and repeat CD rates slowly but steadily increasing. Fetal, early and late neonatal mortality rates decreased by 40–50% from 0.53%, 0.21%, 0.04% in 2000–2001 to 0.29%, 0.12%, 0.02% in 2010, respectively. In 2000–2001, 70.9% of women with a uterine scar had a trial of labor, of which 53.2% had a successful vaginal birth compared with the 64.7% trial of labor and 45.9% success rate in 2010.

**Table 2:** Characteristics of obstetric population ^a^ (including all births, singleton and multiple, from 28+0 to 44+6 gestational weeks), 2000–2010.

Characteristics	2000–01	2002–03	2004–05	2006–07	2008–09	2010
	N = 366,975	N = 365,849	N = 347,036	N = 338,412	N = 345,344	N = 172,343
**Maternal age (years, mean/SD)**	(30.7/4.7)	(30.9/4.8)	(31.0/4.9)	(31.1/4.9)	(31.0/5.0)	(31.0/5.0)
< 20	1.8	1.8	1.6	1.4	1.5	1.4
20–29	39.9	38.0	38.0	39.1	40.1	40.5
30–34	40.5	41.2	39.6	37.4	36.4	36.5
35+	17.8	19.0	20.8	22.1	22.0	21.6
**Ethnicity**						
non-Dutch	18.4	18.3	19.0	20.3	21.5	22.4
**Parity (0/1+)**						
0	47.2	46.4	45.8	45.2	45.7	48.0
**Socioeconomic status**						
3(low)	30.0	30.3	30.6	31.0	31.6	32.3
2(medium)	46.3	46.0	45.6	45.5	45.4	45.2
1(high)	23.7	23.7	23.8	23.4	23.0	22.5
**Multiple gestation (% of all pregnancies)**	2.0	1.9	1.9	1.8	1.7	1.6
**Birthweight (grams, mean/SD)**	(3401/607)	(3415/603)	(3428/604)	(3431/597)	(3434/593)	(3420/586)
< 2500	6.8	6.4	6.3	6.0	5.9	6.0
2500–4000	78.4	78.5	78.0	78.4	78.5	79.4
4000+	14.8	15.1	15.7	15.5	15.6	14.6
**Gestational age (weeks)**						
28–31	0.8	0.8	0.7	0.7	0.7	0.7
32–36	6.2	5.8	5.8	5.8	5.8	5.9
37+	93.0	93.5	93.4	93.5	93.5	93.4
**Fetal presentation**						
non-vertex (%)	6.7	6.4	6.2	5.8	5.5	5.3
**Total Cesarean delivery (%)**	14.0	14.6	14.8	14.8	15.4	16.7
Primary cesarean delivery (%)	11.4	11.9	11.8	11.6	12.0	13.1
Repeat cesarean delivery (%)	2.6	2.7	3.0	3.2	3.4	3.6
**Mortality (%)**						
Fetal	0.53	0.49	0.44	0.38	0.33	0.29
Early neonatal^b^	0.21	0.19	0.17	0.14	0.12	0.12
Late neonatal ^c^	0.04	0.04	0.03	0.03	0.02	0.02
**Trial of labor after previous cesarean delivery**^†^**(%)**	70.9	71.2	71.5	70.0	68.4	64.7
Success rate of VBAC*^†^**(%)**	53.2	54.3	54.3	52.8	50.6	45.9

^a^ Mode of delivery unknown excluded (n = 4,690) from total number of pregnancies with gestational weeks from 28+0 to 44+6

^b^ Early neonatal deaths (0–7 days after live birth)

^c^Late neonatal deaths: postnatal death from 8 to 28 days

^†^including only singleton births

*VBAC: vaginal birth after previous cesarean

Missing data: ethnicity (0.6%), maternal age (0.05%), SES (1.5%), parity (0.01%), fetal position (0.7%)

Some percentages do not add up to hundred because of rounding errors

Table 3 presents the caesarean delivery rates and proportion of total cesarean in subgroups of women with births from 28+0 to 44+6 gestational weeks. The three major contributors for the total CD from 2000 to 2010 were: Groups BR(Breech), NS (nulliparous, spontaneous term labour) and PC (Previous Caesarean). It is interesting to point out that over the study period, the CD rate increased substantially in Group BR (68.0% to 81.8%) and it remains the largest contributor to the overall CD rate even though its relative contribution to the overall CD rate decreased from 27.2% to 21.3%. Likewise, Group TW (Twins/multiplets) had a large increase in CD rate from 37.6% to 47.9% but a decreased contribution to the overall CD rate due to fewer multiple pregnancies. Groups NI (Nulliparous Induced term labour), MI (Multiparous Induced term labour) and MC (Multiparous without uterine scar, term CD before labour) also had a modest increase in the contributions to the overall CD rates. Although the CD rate increased for the Group NS from 8.1% to 9.9%, the contribution to the total CD rate has remained stable (18.7% in 2000–2001 and 18.4% in 2010).

**Table 3:** Caesarean delivery rate and proportion of total cesarean in subgroups of women with births from 28+0 to 44+6 gestational weeks, 2000 to 2010.

Modified Classification		2000–01	2002–03	2004–05	2006–07	2008–09	2010
**NS(1)**	caesarean rate (%)	8.1	8.6	8.6	9.3	9.7	9.9
	proportion of total cesarean	18.7	18.9	18.8	20.2	19.7	18.4
**NI(2a)**	caesarean rate (%)	21.4	22.2	23.6	24.2	24.3	22.4
	proportion of total cesarean	10.2	9.6	9.3	9.7	11.3	12.9
**NC(2b)**	caesarean rate (%)	100	100	100	100	100	100
	proportion of total cesarean	3.5	3.6	3.6	3.3	3.4	3.9
**MS(3)**	caesarean rate (%)	1.7	1.9	1.9	1.8	1.9	2.2
	proportion of total cesarean	4.3	4.6	4.8	4.5	4.3	4.1
**MI(4a)**	caesarean rate (%)	5.3	5.5	5.4	5.6	5.8	6.1
	proportion of total cesarean	2.6	2.4	2.2	2.3	2.6	3.2
**MC(4b)**	caesarean rate (%)	100	100	100	100	100	100
	proportion of total cesarean	5.4	5.7	6.0	6.1	5.8	6.2
**PC(5)**	caesarean rate (%)	43.5	42.4	42.5	43.7	46.7	51.5
	proportion of total cesarean	15.0	15.0	16.4	17.7	18.4	18.1
**BR(6, 7, 9)**	caesarean rate (%)	68.0	77.5	77.3	75.4	78.2	81.8
	proportion of total cesarean	27.2	27.9	26.7	24.2	22.6	21.3
**TW(8)**	caesarean rate (%)	37.6	40.7	40.1	39.0	42.5	47.9
	proportion of total cesarean	5.3	5.4	5.2	4.7	4.8	4.7
**PT(10)**	caesarean rate (%)	21.4	21.0	21.9	22.3	22.4	24.2
	proportion of total cesarean	7.9	6.8	7.1	7.3	7.0	7.1

NS: nulliparous spontaneous; NI: nulliparous induced; NC: nulliparous caesarean; MS: multiparous spontaneous; MI: multiparous induced; MC: multiparous caesarean; PC: previous caesarean; BR: breech; TW: twins; PT: preterm

**Fig 1:**
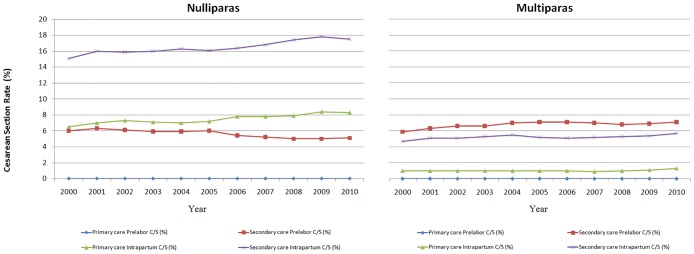
The Cesarean Delivery Rate by level of care at the onset of labor (primary or secondary care) among Singleton Births from 28+0 to 44+6 gestational weeks with Vertex Presentation and no Previous Cesarean Delivery, 2000 to 2010.

Fig 1 shows the CD rate by the level of care at the onset of labor in singleton births from 28+0 to 44+6 gestational weeks with vertex presentation and no previous CD. Among nulliparous and multiparous women, the CD rate showed a modest increase both for women starting labor at primary and secondary care from 2000 to 2010. For nulliparous women in secondary care at the start of labor, there was a considerable rise in the intrapartum CD rate and a small decline in the prelabor CD rate. When the prelabor and intrapartum CD were combined, a small increase was observed.

**Fig 2:**
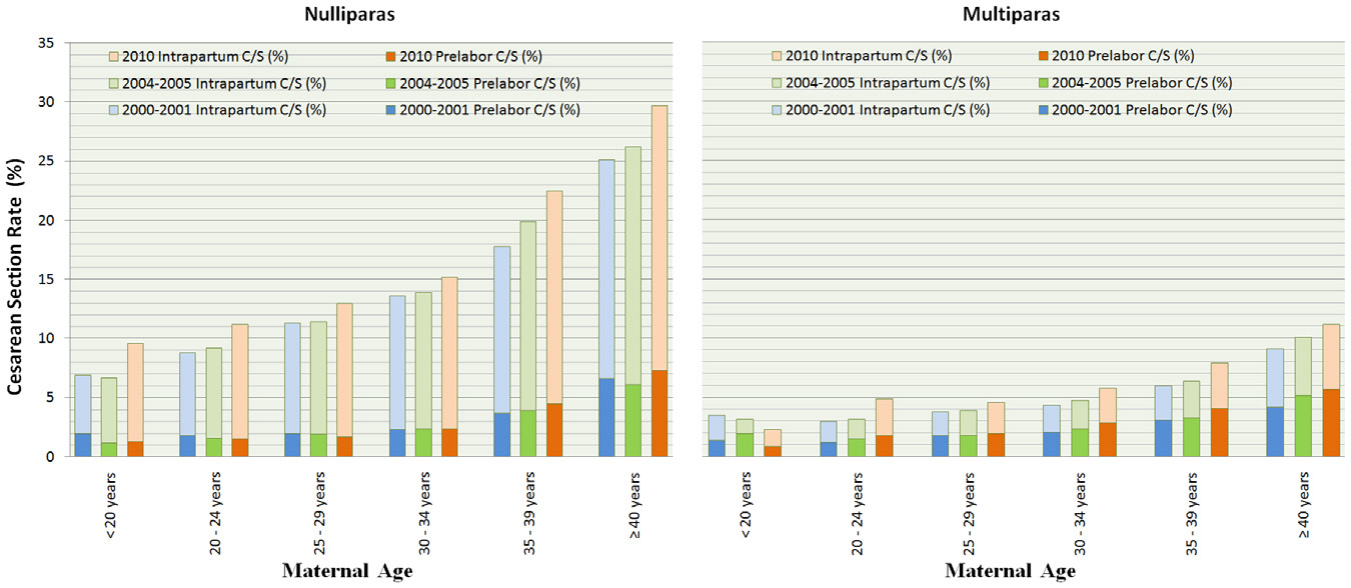
The Intrapartum and Prelabor Cesarean Delivery Rate among Singleton Births from 28+0 to 44+6 gestational weeks with Vertex Presentation and no Previous Cesarean Delivery, by Maternal Age, in 2000–2001, 2004–2005 and 2010.

Fig 2 presents the trends of CD rates by maternal age in 2000–2001, 2004–2005, 2010. Among singleton births from 28+0 to 44+6 gestational weeks with vertex presentation and no previous cesarean delivery, with increasing maternal age, the intrapartum CD, prelabor CD and total CD rate increased in nulliparous women. Most of the increase came from intrapartum CD while the prelabor CD rates remained low. There was a noticeable increase in CD rates over the years even within the same maternal age category.

**Fig 3:**
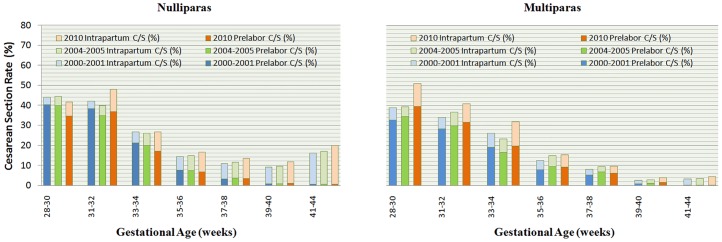
The Intrapartum and Prelabor Cesarean Delivery Rate among Singleton Births from 28+0 to 44+6 gestational weeks with Vertex Presentation and no Previous Cesarean Delivery, by Gestational Age, in 2000–2001, 2004–2005 and 2010.

**Fig 4:**
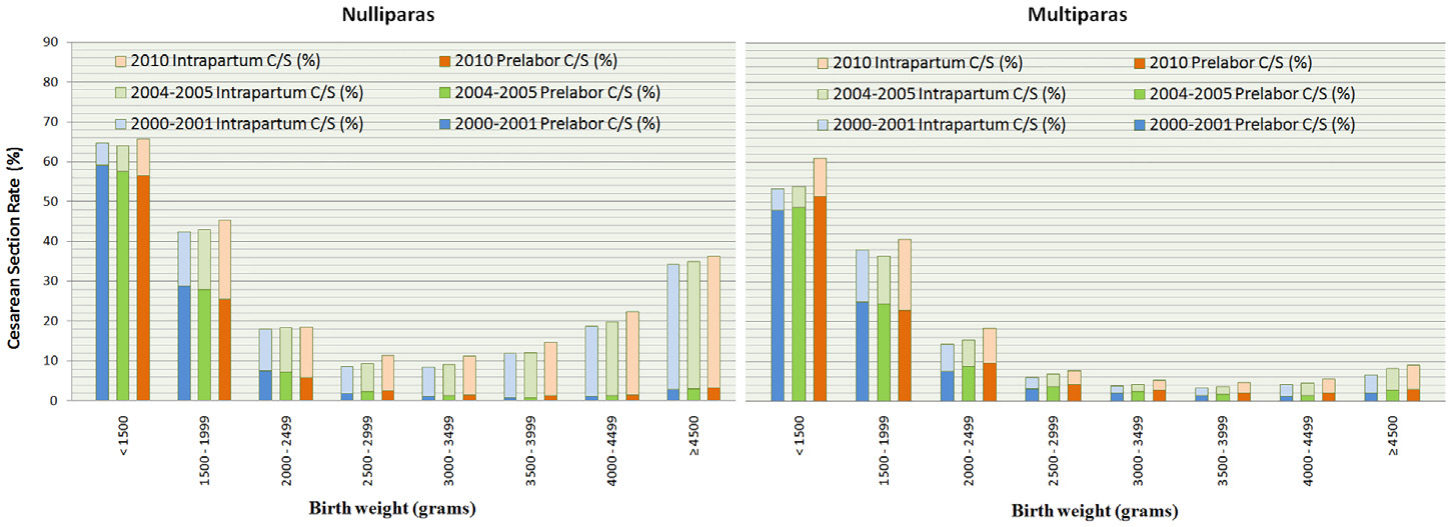
The Intrapartum and Prelabor Cesarean Delivery Rate among Singleton Births from 28+0 to 44+6 gestational weeks with Vertex Presentation and no Previous Cesarean Delivery, by Birth Weight, in 2000–2001, 2004–2005 and 2010.

Figs 3 and 4 show that the CD rates decreased with gestational age until 39–40 weeks and with birthweight until 3000–3499g, particularly for prelabor CD. Within the group of preterm birth and low birthweight (< 2500 grams), the total CD rates were similar among nulliparous and multiparous women and the largest part of the overall CD rate was due to prelabor CD. The CD rates increased more in preterm or low birthweight births than in term and normal weight births.

## Discussion

A recent study suggested that national cesarean delivery rates of up to approximately 19 per 100 live births were associated with lower neonatal mortality among WHO member states[17]. The Netherlands has one of the lowest CD rates among developed countries[9] and still kept it below 19 percent in the past decade. At the same time, perinatal outcomes continue to improve remarkably. By an in-depth examination using the modified Robson classification system, we found that the CD rates increased for all subgroups but changes differed among different groups. For example, the CD rate in pregnancies with abnormal fetal presentation(Group BR) increased substantially from 68.0% to 81.8% during the study. This is consistent with the international trend of a rising planned CD rate for non-vertex births[18,19]. Interestingly, the relative contribution of this group to the overall CD rate actually decreased from 27.2% to 21.3%. Likewise, the CD rate in the multiple gestation (Group TW) has increased from 37.6% to 47.9% from 2000 to 2010 while its relative contribution has decreased from 5.3% to 4.7%. These findings indicate that external cephalic version may be practiced more often, and a reduction in multiple pregnancies. CDs in several groups of term pregnancies with vertex fetuses (NS, MI, MC, PC) became more common, and the groups NI, MI and PC also contributed to the overall rise in CDs.

The CD rate of Group NS is often used for international comparisons[20,21]. In the Netherlands, this group accounted for roughly one-third of all births[12]. It made the second highest contribution to the overall CD rate. The CD rate in this group has risen slightly over the past decades, perhaps partially due to increasing maternal age. The relatively low CD rate in this group also contributes to fewer repeat CDs in the Netherlands compared to other countries[22].

Nevertheless, the CD rate among multiparous women with previous CD (Group PC) increased (from 43.5% to 51.5%), and this group made the third largest contribution to the overall CD rate. Its contribution has been rising (from 15.0% to 18.1%). Despite the fact that the rate of trial of labor after a previous CD is still higher in the Netherlands than in many other countries[23,24], this rate is declining and so is the VBAC success rate. Studies have suggested that several factors such as safety concerns may have contributed to fewer successful VBAC[24–26]. Coupled with a rising CD rate among nulliparous women, the overall CD rate is likely to increase in the Netherlands in the near future.

The Dutch obstetric care system is divided into primary care and secondary care. Primary care is provided by midwives to healthy women with low risk pregnancies. Secondary obstetrician-led care is mainly provided for complicated pregnancies and childbirth. Pregnant women at low risk of complications can choose to give birth at home or in hospital in midwife-led care. If risk factors arise before or during labor, women will be referred to secondary care. More than half of the women in the Netherlands were in primary midwife-led care at the onset of labor[8]. Observational studies in developed countries showed that midwife-led care for low risk women is associated with lower CD rates[27,28]. However, this model in the Netherlands has changed slightly in the past decade. More low risk women in midwife-led care plan their birth in hospital under the care of an independent midwife at the onset of labor[28]. A study showed that in the Netherlands planned hospital birth is associated with higher intervention rates[29]. Also, more women are referred from primary midwife-led care to obstetrician-led care, prenatally as well as intrapartum[29]. Reasons for the higher referral rate including suspected complications during labor and epidural pain relief. When risk factors arise and women need to be referred, their risk for interventions, including a caesarean section, increases likewise.

A major strength of our study is that it was based on a national database with comprehensive information. It gave us a great opportunity to describe the CD trend and to explore practice patterns that have led to a relatively low CD rate in the Netherlands. Nonetheless, we do not have information on BMI and other important risk factors for CD. There may be a presumption that the relatively low CD rate may be due to Dutch women being taller and leaner than women in some other countries. However, a previous report showed that women in other countries had a similar BMI as Dutch women, but a much higher CD rate[30]. Thus, the BMI distribution of pregnant women may not be a critical factor for a low CD rate in a country.

Another limitation of our data lies in the fact that in registration data some information will be missing or misclassified. For example, some multiparous women have no record of a previous CD in the PR, leading to a misclassification of repeat CD to primary CD[12]. A recent study suggested that 27.4% of multiparous women with a history of CD had no record of this in the PR[31]. This may lead to an overestimation of the CD rate in the MS, MI and MC groups and underestimation of the CD rate in the PC group. The findings on the CD rate and relative contribution to the overall CD rate of this group may be inaccurate. Nonetheless, the total CD rate in the current pregnancy is not affected by this misclassification. And assuming that the underreporting of previous CD has been more or less constant during the study years, the change of CD rate, therefore, may have not been affected to the same degree as the absolute CD rate in this group at a particular year.

It is worth noting that although our study focuses on the changes of CS rates in subgroups over the past decade, we found that the overall CD rate increased slightly duringthe period of 2000to 2010 with the primary and repeat CD rates slowly but steadily increasing. Fetal, early and late neonatal mortality rates decreased by 40–50% from 0.53%, 0.21%, 0.04% in 2000–2001 to 0.29%, 0.12%, 0.02% in 2010, respectively. These mortality rates rank in the middle of the European countries[9], suggesting that the Dutch mothers and babies are doing well.

In summary, our analysis of CD rates in the Netherlands suggests that to keep a low, stable CD rate, a very critical attitude towards the CD in nulliparous women is the key not only for the current overall CD rate but also for the future rate[32]. The doctor deciding to perform the first CD should feel responsible for/take into account potential long term complications of that decision in future pregnancies. At the same time, encouraging a trial of labor in women with a previous uterine scar and improving the success rate of VBAC are important factors in keeping the CD rate low, as well as performing external cephalic versions to prevent breech presentation in term births.

## References

[1] Joseph KS, Young DC, Dodds L, O'Connell CM, Allen VM, Chandra S, et al Changes in maternal characteristics and obstetric practice and recent increases in primary cesarean delivery. *Obstet Gynecol* 2003;102:791–800. 14551010 10.1016/s0029-7844(03)00620-3

[2] Macones GA. Clinical outcomes in VBAC attempts: what to say to patients? *Am J Obstet Gynecol* 2008;199:1–2. 10.1016/j.ajog.2008.03.040 18585519

[3] Habiba M, Kaminski M, Da Fré M, Marsal K, Bleker O, Librero J, et al Cesarean section of request: a comparison of obstetricians’ attitudes in eight European countries. *BJOG* 2006;113:647–56. 16709207 10.1111/j.1471-0528.2006.00933.x

[4] Porreco RP, Thorp JA. The caesarean birth epidemic: trends, causes, and solutions. *Am J ObstetGynecol* 1997;175: 369–74. 10.1016/s0002-9378(96)70148-5 8765255

[5] Murthy K, Grobman WA, Lee TA, Holl JL. Association between rising professional liability insurance premiums and primary cesarean delivery rates. *Obste tGynecol* 2007;110:1264–9. 10.1097/01.AOG.0000287294.89148.23 18055719

[6] Elferink-Stinkens PM, Brand R, Van Hemel OJ. Trends in caesarean section rates among high- and medium-risk pregnancies in the Netherlands 1983–1992. *European Journal of Obstetrics*, *Gynecology and Reproductive Biology* 1995;59: 159–67. 10.1016/0028-2243(95)02050-3 7657010

[7] Kwee A, Elferink-Stinkens PM, Reuwer PJHM, Bruinse HW. Trends in obstetric interventions in the Dutch obstetrical care system in the period 1993–2002. *European Journal of Obstetrics*, *Gynecology and Reproductive Biology* 2007;132:70–75. 10.1016/j.ejogrb.2006.06.018 16884843

[8] StichtingPerinataleRegistratie Nederland, 2011b. Perinatal Care in the Netherlands 2008. StichtingPerinataleRegistratie Nederland, Utrecht.

[9] EURO-PERISTAT. The European Perinatal Health Report (http://www.europeristat.com/reports/european-perinatal-health-report-2010.html) (last accessed August 2015).

[10] Robson MS. Classification of caesarean sections. *Fetal Matern Med Rev* 2001;12: 23–39.

[11] Robson MS. Can we reduce the caesarean section rate? *Best Pract Res ClinObstetGynaecol* 2001;15: 179–94. 10.1053/beog.2000.0156 11359322

[12] Zhang J, Geerts C, Hukkelhoven C, Offerhaus P, Zwart J, de Jonge A. Caesarean section rates in subgroups of Dutch women and perinatal outcomes. *BJOG* 2015 7 22 10.1111/1471-0528.13520 26216434

[13] Betran AP, Vindevoghel N, Souza JP, Gülmezoglu AM, Torloni MR.A systematic review of the Robson classification for caesareansection: what works, doesn’t work and how to improve it. *PLoS ONE* 2014;9:e97769 10.1371/journal.pone.0097769 24892928 PMC4043665

[14] Meray N, Reitsma JB, Ravelli AC, Bonsel GJ. Probabilistic record linkage is a valid and transparent tool to combine databases without a patient identification number. *J ClinEpidemiol* 2007;60:883–91. 10.1016/j.jclinepi.2006.11.021 17689804

[15] Offerhaus PM, Hukkelhoven CW, de Jonge A, van der Palde Bruin KM, Scheepers PL, LagroJanssen AL. Persisting rise in referrals during labor in primary midwife-led care in the Netherlands.*Birth* 2013; 40:192–201. 10.1111/birt.12055 24635504

[16] De Jonge A, Van der Goes BY, Ravelli AC, Amelink-Verburg MP, Mol BW, Nijhuis JG, et al Perinatal mortality and morbidity in a nationwide cohort of 529,688 low-risk planned home and hospital births. *BJOG* 2009; 116: 1177–84. 10.1111/j.1471-0528.2009.02175.x 19624439

[17] Molina G, Weiser TG, Lipsitz SR, Esquivel MM, Uribe-Leitz T, Azad T, et al Relationship Between Cesarean Delivery Rate and Maternal and Neonatal Mortality.*JAMA* 2015; 314:2263–70. 10.1001/jama.2015.15553 26624825

[18] Hofmeyr GJ, Hannah M. Planned caesarean section for term breech delivery. *Cochrane Database Syst Rev* 2003; (3): CD000166 12917886 10.1002/14651858.CD000166

[19] Hannah ME, Hannah WJ, Hewson SA, Hodnett ED, Saigal S, Willan AR.Planned caesarean section versus planned vaginalbirth for breech presentation at term: a randomisedmulticentre trial. Term Breech Trial Collaborative Group.*Lancet* 2000; 356: 1375–1383. 11052579 10.1016/s0140-6736(00)02840-3

[20] Brennan DJ, Robson MS, Murphy M, O’Herlihy C. Comparative analysis of international cesarean delivery rates using 10-group classification identifies significant variation in spontaneous labor. *Am J ObstetGynecol* 2009;201:308.e1–308.e8. 10.1016/j.ajog.2009.06.021 19733283

[21] Brennan DJ, Murphy M, Robson MS, O’Herlihy C. The singleton, cephalic, nulliparous woman after 36 weeks of gestation: contribution to overall cesarean delivery rates. *Obstet Gynecol* 2011; 117: 273–79. 10.1097/AOG.0b013e318204521a 21252739

[22] Vogel JP, Betrán AP, Vindevoghel N, Souza JP, Torloni MR, Zhang J, et al Use of the Robson classification to assess caesarean section trends in 21 countries: a secondary analysis of two WHO multicountry surveys. *Lancet Glob Health* 2015; 3: e260–70. 10.1016/S2214-109X(15)70094-X 25866355

[23] Howell S, Johnston T, Macleod SL.Trends and determinants of caesarean section births in Queensland, 1997–2006. *Aust N Z J ObstetGynaecol* 2009; 49:606–11. 10.1111/j.1479-828X.2009.01100.x 20070708

[24] Guise J-M, Berlin M, McDonagh M, Osterweil P, Chan B, Helfand M. Safety of vaginal birth after caesarean: a systematic review.*ObstetGynecol* 2004; 103: 420–29. 10.1097/01.AOG.0000116259.41678.f1 14990401

[25] Dodd JM, Crowther CA, Huertas E, Guise JM, Horey D. Planned elective repeat caesarean section versus planned vaginal birth for women with a previous caesarean birth. *Cochrane Database Syst Rev* 2013; 12: CD004224 10.1002/14651858.CD004224.pub3 15495090

[26] Macones GA. Clinical outcomes in VBAC attempts: what to say to patients? *Am J Obstet Gynecol* 2008;199:1–2. 10.1016/j.ajog.2008.03.040 18585519

[27] Janssen PA, Saxell L, Page LA, Klein MC, Liston RM, Lee SK. Outcomes of planned home birth with registered midwife versus planned hospital birth with midwife or physician. *Canadian Medical Association Journal* 2009:181,377–83. 10.1503/cmaj.081869 19720688 PMC2742137

[28] Birthplace in England Collaborative Group, 2011 Perinatal and maternal outcomes by planned place of birth for healthy women with low risk pregnancies: the Birthplace in England national prospective cohort study. *BMJ* 343, d7400 10.1136/bmj.d7400 22117057 PMC3223531

[29] Offerhaus PM, de Jonge A, van der Pal-de Bruin KM, Hukkelhoven CW, Scheepers PL, Lagro-Janssen AL. Change in primary midwife-led care in the Netherlands in 2000–2008: A descriptive study of caesarean sections and other interventions among 807,437 low risk births. *Midwifery* 2015; 31: 648–54. 26203475 10.1016/j.midw.2015.01.013

[30] Data and Information on Women’s Health in the European Union. Dresden, Germany: Faculty of Medicine Carl Gustav Carus, Research Association Public Health Saxony and Saxony-Anhalt, Technische Universität Dresden, 2009. ISBN-978-92-79-13659-7.

[31] de Jonge A, Mesman JA, Manniën J, Zwart JJ, van Dillen J, van Roosmalen J. Severe adverse maternal outcomes among low risk women with planned home versus hospital births in the Netherlands: nationwide cohort study. *BMJ* 2013; 346: f3263 10.1136/bmj.f3263 23766482 PMC3685517

[32] Spong CY. Prevention of the first cesarean delivery. *Obstet Gynecol Clin North Am* 2015; 42: 377–80. 10.1016/j.ogc.2015.01.010 26002173 PMC4441950

